# The Prince of Wales's Hospital Fund for London

**Published:** 1897-06-05

**Authors:** 


### The Institutional Workshop.

### THE PRINCE OF WALES'S HOSPITAL FUND FOR LONDON.

### The Hospital Stamp.

The visit of T.R.H. the Prince and Princess of Wales and Princess Victoria to the works of Messrs. De la Rue and Co., Bunhill Row, to see the Hospital Stamps printed, has caused general interest amongst all classes in London, and, indeed, throughout the country. This visit was due to the great personal interest which the Prince and Princess take in the success of the stamp scheme, and was an act of kindness on their part at the busiest season of the year which all hospital people will no doubt remember with pleasure and gratitude. We give an account of the inspection made by their Royal Highnesses further on, but may here specially emphasise the genuine interest shown by the Prince and Princess in all they saw at Messrs. De la Rue's most interesting manufactory. Indeed H.R.H. the Princess was so impressed with one of the machines for perforating postal letter cards that she requested permission to work it herself. Both the Princess of Wales and Princess Victoria perforated a number of postal letter cards, and throughout their inspection they showed the greatest interest in the girls employed, the machines of which they had charge, and the little personal decorations which many of the girls had added to their work tables, some of the pictures being quaint and curious. This Royal visit to Bunhill Row ought to make everybody acquainted with the hospital stamps, so that none will be able to complain should they find it impossible to purchase one except at a premium, owing to their dilatoriness in not giving an order through their bookseller or newsagent before the end of the first week in June.

### The History of the Stamps.

Some controversy having arisen as to the origin of the stamps, it may be well to give a brief history of the matter. At the end of 1896 it became necessary to arrive at some decision as to what form of commemoration of the 60th year of the Queen's reign Londoners should be invited to adopt. Out of many hundreds of proposals it was ultimately determined to make a supreme effort to permanently benefit the hospitals. It was seen that a large sum of money would be required —from £70,000 to £100,000 a year—and various plans for raising this money were formulated. In addition to the subscribers of one guinea a year and upwards, it was always intended to reach, if possible, the humbler residents in the metropolis, who would give their one shilling a year and upwards. It was felt, however, that it would be undesirable to attempt to get at the shilling constituency until the wealthier people had first been approached. The *Daily Telegraph* started a shilling collection at the outset, however, independently of the Council of the Prince of Wales's Fund, and this they have pushed in all directions, so that the *Daily Telegraph* appeals were circulated amongst all classes before any appeal was issued by the Prince's Fund Council. Although the well-to-do classes were approached first by the authorities of the Fund, even before the first meeting at Marlborough House a plan had been formulated for raising shilling subscriptions, a plan which would prevent fraud, and ensure the collection of the money at the smallest possible cost. This plan for raising subscriptions of from one to ten shillings per annum involved a communication to the authorities of the Post Office, inviting them to permit such subscriptions to be received at the Post Office Savings Banks, or at the post offices in London. The first proposal would have obviated the necessity of issuing a stamp, but the second necessitated such an issue, to enable the Post Office to receive and account for the money without difficulty. After weeks of negotiations the Post Office and the Government declined to have anything to do with these collections, but it will be seen that before any public announcement was made, or any paragraph appeared in any newspaper, it had been determined that the original plan involved, in certain eventualities, the issue of a stamp to facilitate the collection of small subscriptions. The first meeting of the Prince of Wales's Fund was held at Marlborough House on January 28th, and on February 8th the *Daily Mail* threw out a suggestion for the issue of a stamp in connection with the Fund. On February 9th the *Daily Mail* published a suggestion from a correspondent that the stamps should be sold by the Post Office, and that their face value should be 1s. and 10s. respectively. On February 13th the *Spectator* published an article entitled "The Mechanism of Philanthropy," in the course of which they advocated the issue through the General Post Office of stamps for 1s. and 2s. 6d., with the word "Charity" stamped across them. The *Spectator's* scheme was well thought out, and was also very complete in its details. When the Post Office and the Government declined to have anything to do with the issue of stamps in connection with the Prince of Wales's Fund, it became necessary to make other arrangements, and the present plan was thought out and carried through, with the assistance of Mr. Thomas De La Rue, who has rendered yeoman service in the matter, Mr. Purcell, C.B., and Mr. G. H. Miles, of Messrs. Simpkin, Marshall, and Co. (Limited), who have intimate relations with the booksellers, stationers, newsagents, and stamp dealers throughout the country and the colonies. It will thus be seen that the idea of a stamp was part of the Prince of Wales's original scheme for helping the hospitals; that the *Daily Mail* was the first newspaper to call public attention to the desirability of issuing such stamps; whilst the *Spectator* was the journal which thought out a workable scheme, and which, in fact, suggested the face values of the two classes of stamps which have ultimately been issued. We certainly hope, although the demand for the stamps is already phenomenal, that both the *Spectator* and the *Daily Mail* will continue to take the hospital stamps into their keeping and that they will use their powerful influence to awaken all classes of the community, and especially the working classes, to the facilities which these stamps afford to them for becoming regular subscribers of small sums to the hospitals in the readiest and least costly manner.

